# Adipose sirtuin 6 drives macrophage polarization toward M2 through IL-4 production and maintains systemic insulin sensitivity in mice and humans

**DOI:** 10.1038/s12276-019-0256-9

**Published:** 2019-05-21

**Authors:** Mi-Young Song, Sang Hoon Kim, Ga-Hee Ryoo, Mi-Kyung Kim, Hye-Na Cha, So-Young Park, Hong Pil Hwang, Hee Chul Yu, Eun Ju Bae, Byung-Hyun Park

**Affiliations:** 1 Department of Biochemistry and Molecular Biology, Chonbuk National University Medical School, Jeonju, Jeonbuk 54896 Republic of Korea; 2 Department of Surgery, Chonbuk National University Medical School, Jeonju, Jeonbuk 54896 Republic of Korea; 3 Research Institute of Dong-A ST Co. Ltd., Yongin, Gyeonggi 17073 Republic of Korea; 4 Department of Physiology, College of Medicine, Yeungnam University, Daegu, 42415 Republic of Korea; 5 College of Pharmacy, Woosuk University, Wanju, Jeonbuk 55338 Republic of Korea

**Keywords:** Obesity, Translational research, Type 2 diabetes

## Abstract

Adipose tissue inflammation is a reproducible feature of obesity and obesity-linked insulin resistance. Although sirtuin 6 (Sirt6) deficiency has previously been implicated in diet-induced obesity and systemic insulin resistance, the adipocyte-specific role of Sirt6 in the regulation of adipose tissue inflammation and systemic metabolic dysfunction in mice fed normal chow and in humans remains elusive. Here, using *Adipoq-Cre*-mediated adipocyte-specific Sirt6 knockout (aS6KO) mice, we explored whether adipocyte Sirt6 inhibits adipose tissue inflammation and its underlying mechanism. aS6KO mice fed normal chow gained more body weight and fat mass than wild-type mice and exhibited glucose intolerance and systemic insulin resistance. Measurement of plasma and tissue cytokines and flow cytometric analysis of adipose stromal vascular cells indicated a decrease in alternatively activated M2 macrophages in the adipose tissue of aS6KO mice. Mechanistically, Sirt6 regulated the expression of the canonical type 2 cytokine IL-4 by adipocytes in a cell autonomous manner, which in turn affects M2 macrophage polarization. Consistent with animal experimental data, the degree of obesity and insulin resistance demonstrated by the body mass index, fasting blood glucose and HbA1c correlated negatively with the expression of Sirt6 in human visceral fat tissues. Collectively, these results suggest that adipocyte Sirt6 regulates body weight gain and insulin sensitivity independent of diet, and the increased IL-4 production by Sirt6 and resultant M2 polarization of adipose tissue macrophages may attenuate proinflammatory responses in adipose tissue.

## Obesity and diabetes: A control protein in fat

A protein in adipose tissue (composed of fat cells) helps protect against inflammation and the development of resistance to insulin that develops in obesity and can lead to type 2 diabetes. Researchers in South Korea, led by Eun Ju Bae at Woosuk University, Wanju, and Byung-Hyun Park at Chonbuk National University, Jeonju, investigated the role of Sirt6 in mice and in human adipose tissue. Deleting the mouse gene that codes for Sirt6 in adipocytes promoted the impaired response to insulin and associated increase in blood glucose levels that are two key aspects of diabetes. Changes in biochemical signaling pathways controlling immune cells called macrophages were implicated in these effects and suggest an anti-inflammatory role for Sirt6. Analysis of human adipose tissue supported these findings. The research will help understand how obesity promotes type 2 diabetes.

## Introduction

Obesity and type 2 diabetes are considered to be a chronic low-grade inflammatory state termed “meta-inflammation”^1^. Histologically, the inflammation of white adipose tissue (WAT) is accompanied by increased accumulation of a variety of immune cells, mainly macrophages^2^. Adipose tissue macrophages (ATMs) cluster around and clear dead adipocytes, forming a crown-like structure (CLS) that is a distinctive feature of low-grade inflammation in WAT, and CLS density correlates with the degree of obesity^3^. Although hypertrophied adipocytes themselves secrete cytokines and chemokines^4^, ATMs are considered the primary source of adipose tissue-derived proinflammatory mediators^5^. ATMs are heterogeneous and remarkably plastic and are generally present as two major subpopulations: classically activated M1 macrophages and alternatively activated M2 macrophages. Whereas anti-inflammatory M2 macrophages are predominant in WAT in lean states, high-fat diet (HFD) feeding triggers the infiltration of proinflammatory M1 macrophages^6,7^. These M1 macrophages release proinflammatory cytokines that cause metabolic derangement.

Sirtuins (Sirt1-7) are NAD^+^-dependent deacetylases that are important for coordination of metabolic homeostasis^8^. Sirt6 is unique in its localization to the nucleus and functions as a deacetylase of both acetyl- and fatty acyl-groups and ADP-ribosyltransferase^9,10^. Through its function as a deacetylase, this enzyme modulates the histone acetylation state and thus regulates the expression of target genes^11–13^. This protein also directly regulates the activities of various non-histone proteins by lysine deacetylation^14–18^. *Sirt6* transgenic mice show reduced fat mass, lower LDL cholesterol and triglyceride levels, and improved glucose tolerance, and these effects are mediated by suppression of PPARγ target genes^19^. Conversely, adipocyte-specific Sirt6 deletion causes increases in body weight and fat mass compared with wild-type (WT) mice^20,21^. Consistent with these reports, Sirt6 expression is suppressed in adipose tissues of high-fat-fed mice and obese humans^20,22^. Interestingly, deletion of adipocyte Sirt6 accelerates M1 macrophage infiltration into WAT with no change in the M2 macrophage population and promotes systemic insulin resistance in mice fed a HFD^19^. These findings suggest that Sirt6 couples adipose tissue inflammation to host glucose homeostasis through regulation of macrophage polarization under high-calorie diet conditions. However, it is not clear how adipocyte Sirt6 deficiency attracts a specific macrophage subtype into the WAT and whether this phenomenon is also observed under normal chow diet (NCD) feeding conditions. To investigate this, we generated adipocyte-specific Sirt6 knockout (*Sirt6*^*fl/fl*^:*Adipoq-Cre*, aS6KO) mice and analyzed these mice after they were fed a NCD. The animal study demonstrated that Sirt6 deficiency in adipocytes leads to increased accumulation of macrophages in WAT and systemic insulin resistance under NCD, which recapitulates the phenotypes observed in HFD-fed or genetically obese mice. Mechanistically, Sirt6 in adipocytes increases the production of the type 2 cytokine IL-4 to drive M2 polarization in a paracrine manner. Tissue analysis of visceral fat from humans showed a negative correlation between Sirt6 and either adiposity parameters (body mass index and waist circumference) or insulin sensitivity parameters (fasting blood glucose and HbA1c). Hence, this study establishes Sirt6 as a key determinant of adipose macrophage content and type in mice and humans.

## Materials and Methods

### Animals

*Sirt6*^*flox/flox*^ mice (B6;129-*Sirt6*^*tm1Ygu*^/J) and *Adipoq-Cre* mice (B6. FVB-Tg(*Adipoq-cre*)1Evdr/J) were obtained from the Jackson Laboratory (Bar Harbor, ME, USA). *Sirt6*^*fl/fl*^ and *Adipoq-Cre* mice were crossed to obtain aSirt6 KO mice. For genotyping, tail tips were incubated with STE buffer (100 mM Tris, 5 mM EDTA, 0.2% SDS, and 200 mM NaCl, pH 7.4) and 0.25 mg/ml proteinase K for 6 h at 55 °C and submitted to a two-step PCR with Taq polymerase (Clontech, Mountain View, CA, USA) and specific forward (5′-AGTGAGGGGCTAATGGGAAC-3′) and reverse (5′-AACCCACCTCTCTCCCCTAA-3′) primers. Amplification of a 453-bp band confirmed the *Sirt6* genotype.

### Body fat percentage

The body fat percentage was determined using a Bruker Minispec mq 7.5 NMR analyzer (Bruker Optics, Ettlingen, Germany) as described previously^23^.

### Glucose and insulin tolerance tests

aS6KO mice and age-matched WT littermates older than 6 weeks were fed a standard laboratory NCD (Research Diet, New Brunswick, NJ, USA) ad libitum. At the age of 16 weeks, the intraperitoneal glucose tolerance test (GTT) and insulin tolerance test (ITT) were performed over a 3-day interval. After 12 h of fasting, the mice received a glucose solution intraperitoneally at a dose of 1 g/kg body weight. The glucose concentration was evaluated in blood samples collected from the tail at 0 (baseline), 15, 30, 60, 90, and 120 min after glucose injection. For ITT, after a 6-h fast, glucose levels were likewise measured from the tail vein after intraperitoneal injection with 0.75 units/kg body weight of human insulin (Sigma-Aldrich, St Louis, MO, USA). In vivo glucose utilization and metabolic rate were measured by the hyperinsulinemic-euglycemic clamp and by the indirect calorimetry, respectively, as described previously^24^. All experimental procedures were approved by the Institutional Animal Care and Use Committee of Chonbuk National University (permit number: CBNU-2017-0117).

### Human tissues

Human abdominal fat tissues close to the bladder were removed during elective or emergency kidney transplantation (*n* = 44) at the Surgery Unit of Chonbuk National University Hospital (Jeonju, Korea). The tissues were rinsed with PBS and immediately stored at −70 °C. The demographic and clinical characteristics of 44 patients enrolled in this study are summarized in Table S1. All patients provided written informed consent, and the study was approved by the Institutional Review Board of Chonbuk National University Hospital (permit number: CUH 2017-03-026).

### Histology

Abdominal fat tissues were immediately placed in fixative (10% formalin solution in 0.1 M PBS). Histological sections (6 μm) were cut from formalin-fixed paraffin-embedded tissue blocks. Tissue sections were stained with hematoxylin-eosin (H&E) under standard conditions. Immunohistochemical staining was performed using the DAKO Envision system (DAKO, Carpinteria, CA, USA). Sections were immunostained with antibodies against F4/80 (Abcam, Cambridge, UK) and perilipin (Fizerald, Acton, MA, USA). Peroxidase activity was detected with 3-amino-9-ethyl carbazole. The adipocyte area in selected fat tissue sections was measured using iSolution DT 36 software (Carl Zeiss, Oberkochen, Germany). The number of F4/80^+^ cells and CLS was counted in five different high-power fields from each section.

### Biochemical analysis

IL-4, IL-13, TNF-α (eBioscience, San Diego, CA, USA), insulin, leptin, adiponectin (ALPCO, Salem, NH, USA), and CCL2 (Peprotech, Rocky Hill, NJ, USA) were measured using specific ELISA kits. Plasma levels of glycerol (Sigma-Aldrich) and non-esterified fatty acid (Biovision, Milpitas, CA, USA) were measured using commercially available kits.

### Western blotting

Tissue homogenates or cell lysates (20 μg) were separated by 10% SDS-PAGE and transferred to PVDF membranes. After blocking with 5% skim milk, the blot was probed with primary antibodies against Sirt6, Akt, p-Akt (Ser473), p-HSL (Ser660), p-HSL (Ser563), HSL, ATGL, p-AS160 (Thr642), p-GSK3β (Ser9), FoxO1, p-FoxO1 (Thr24), p-perilipin-1 (Cell Signaling, Beverly, MA, USA), p-ATGL (Ser406), perilipin-1 (Abcam, Cambridge, UK), HSP90 (Enzo Life Sciences, Plymouth Meeting, PA, USA), and AS160 (Millipore, Danvers, MA, USA). Immunoreactive bands were detected with a Las-4000 imager (GE Healthcare Life Science, Pittsburgh, PA, USA).

### RNA isolation and real-time quantitative RT-PCR (qPCR)

Total RNA was extracted from frozen liver tissue using an RNA Iso kit (TaKaRa, Tokyo, Japan). First-strand cDNA was generated using the random hexamer primer provided in the first-strand cDNA synthesis kit (Applied Biosystems, Foster City, CA, USA). Specific primers for each gene (Table S2) were designed using qPrimerDepot (http://mouseprimerdepot.nci.nih.gov). qPCR reactions were performed in a final volume of 10 μl containing 10 ng of reverse-transcribed total RNA, 200 nM of forward and reverse primers and PCR master mix. qPCR was performed in 384-well plates using an ABI Prism 7900HT Sequence Detection System (Applied Biosystems).

### Flow cytometric analysis

Stromal vascular cells (SVCs) from epididymal fat pads were isolated as described previously^24^. SVCs were incubated in FACS buffer containing 2% FBS with Fc Block (BD Biosciences, San Jose, CA, USA) for 30 min at 4 °C prior to staining with antibodies against F4/80 (1 μg/ml), CD11b (0.4 μg/ml), or CD11c (0.4 μg/ml) for 30 min at 4 °C. Primary antibodies were obtained from BD Biosciences. Stained cells were gently washed three times and resuspended in FACS buffer. SVCs were analyzed using a FACSCalibur™ instrument (BD Biosciences). Unstained, single stained, and fluorescence minus one control were used to set compensation and gates.

### Adipocyte differentiation of SVCs

SVCs from epididymal fat pads of WT and aS6KO mice were isolated and differentiated into mature adipocytes by culture in DMEM medium supplemented with 10% FBS, 0.5 mM isobutyl-1-methylxanthine, 1 μM dexamethasone, 10 μg/ml insulin, and 1 μM rosiglitazone. At the end of day 8, mature adipocytes were incubated in 10% FBS containing DMEM for 48 h, and conditioned medium (CM) was collected.

### Macrophage polarization

To generate bone marrow macrophages (BMMs), bone marrow cells were flushed out from the tibias and femurs of WT mice. Single-cell suspensions of total bone marrow were cultured in α-MEM supplemented with 30% L929 cell CM as a source of M-CSF. For M1 or M2 polarization, BMMs were incubated in CM from WT or KO SVC-derived adipocytes.

### Statistical analysis

Data are expressed as the mean ± standard error of the mean (SEM). Statistical comparisons were made using one-way analysis of variance followed by Fisher’s post hoc analysis. The significance of differences between groups was determined using Student’s unpaired *t*-test. A *p* value <0.05 was considered significant.

## Results

### Adipocyte-specific Sirt6 deficiency increases fat mass

To investigate the physiological role of Sirt6 in adipose tissue, we first evaluated the relative level of Sirt6 in adipocytes and macrophage-containing SVCs obtained from eWAT from mice fed a NCD. Sirt6 protein was predominantly detected in the adipocyte fraction with only marginally detectable levels in SVCs (Fig. S1A). In addition, we observed a marked decrease in Sirt6 expression in eWAT from 16-week HFD-fed mice compared with NCD-fed mice (Fig. S1B), suggesting a possible contribution of adipose Sirt6 to weight gain and/or insulin resistance. To provide more direct evidence supporting this hypothesis, we generated mice lacking Sirt6 in adipocytes by mating Sirt6 floxed mice (*Sirt6*^*fl/fl*^) with *Adipoq-Cre* mice (Fig. S1C). Genotyping, RT-PCR, and western blotting confirmed the efficient and specific deletion of Sirt6 in brown adipose tissue (BAT), inguinal WAT (iWAT), and epididymal WAT (eWAT) but not in other tissues, including liver and skeletal muscle (Fig. S1D–F).

**Fig. 1:**
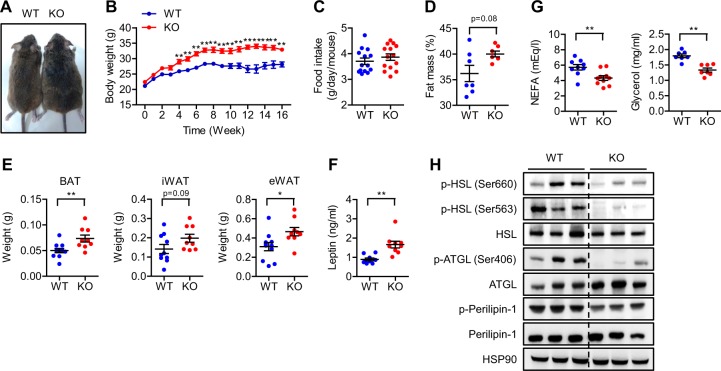
Increased fat accumulation in aS6KO mice. **a** Representative photograph of WT and aS6KO siblings at 16 weeks of age. **b**, **c** Weight gain and food intake of WT and aS6KO mice after feeding with the normal chow diet (*n* = 9–10 per group). **d** Fat content in mice measured by an NMR analyzer (*n* = 7 per group). **e** Quantification of different fat depots in WT and aS6KO mice (*n* = 14 per group). **f**, **g** Plasma levels of leptin, non-esterified fatty acid (NEFA), and glycerol measured using a specific assay kit (*n* = 7–10 per group). **h** Protein levels of lipolysis-related proteins in eWAT (*n* = 3 per group). Values are the mean ± SEM. *p < 0.05 and **p < 0.01 vs. WT

aS6KO mice were born at the expected Mendelian ratios and were indistinguishable from their WT littermates. On a NCD, aS6KO mice gained more weight than their WT littermates with similar food intakes (Fig. 1a–c). We observed the same trend when we measured body fat mass by a nuclear magnetic resonance (NMR) analyzer (Fig. 1d). aS6KO mice showed a significant increase in BAT and eWAT (Fig. 1e). Plasma leptin levels were well correlated with fat mass (Fig. 1f). Consistent with a previous report^20^, plasma levels of glycerol and non-esterified fatty acids and tissue levels of key lipolytic proteins, such as hormone-sensitive lipase (HSL), adipose triglyceride lipase (ATGL) and perilipin, were significantly decreased in aS6KO mice (Fig. 1g, h), indicating decreased lipolysis in aS6KO mice.

**Fig. 2:**
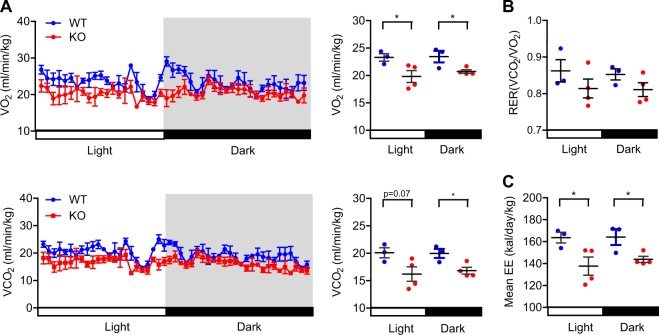
Decreased energy expenditure in aS6KO mice. Indirect calorimetry was performed using an 8-chamber Oxymax system. Mice were acclimatized to cages for 24 h, and data were collected for an additional 24 h (*n* = 3–4 per group). **a** Twenty-four hour O_2_ consumption rates (VO_2_) and CO_2_ production rates (VCO_2_) in normal chow-fed mice. **b** Respiratory exchange ratio (RER) calculated as the volume of CO_2_ versus the volume of O_2_. **c** Twenty-four-hour average energy expenditure (EE). Values are the mean ± SEM. *p < 0.05 vs. WT

To assess how Sirt6 deficiency affects weight gain, we performed metabolic cage studies. aS6KO mice had decreased VO_2_ and VCO_2_ and maintained a lower respiratory exchange ratio (RER, VCO_2_/VO_2_) compared with WT mice (Fig. 2a, b). Energy expenditure was also significantly decreased in the aS6KO mice during both the dark and light phases (Fig. 2c), suggesting that Sirt6 deficiency increases fat mass by suppressing both energy expenditure and lipolysis.

### Adipocyte-specific Sirt6 deficiency induces systemic insulin resistance

**Fig. 3:**
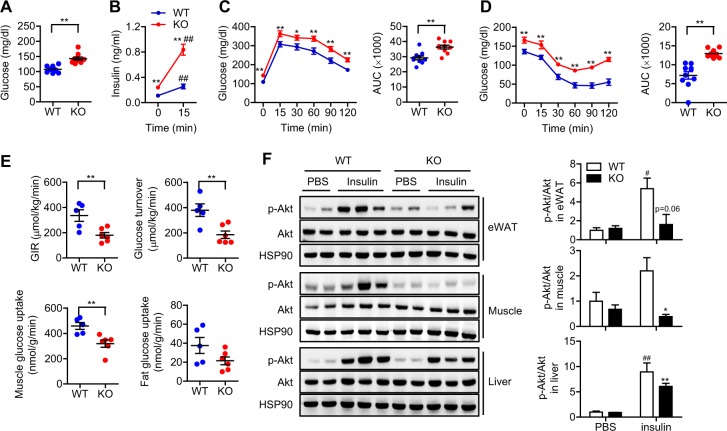
Development of systemic insulin resistance in aS6KO mice. WT or aS6KO mice were fed a NCD for 16 weeks. **a** Fasting glucose levels (*n* = 10 per group), **b** basal and stimulated nsulin levels (*n* = 8–9 per group), **c** glucose concentrations during the intraperitoneal glucose tolerance test (*n* = 9–10 per group), and **d** glucose concentrations during the insulin tolerance test (*n* = 9–10 per group) were measured. Areas under the curve were compared. **e** The glucose infusion rate (GIR), whole-body glucose turnover, and glucose uptake levels in the soleus muscle and eWAT were determined during the hyperinsulinemic-euglycemic clamp test (*n* = 5–6 per group). **f** Adipose tissue-, skeletal muscle-, and liver-specific insulin sensitivity was measured by assessing the level of insulin-stimulated Akt phosphorylation. WT and aS6KO mice were fasted for 6 h and then injected with insulin (0.75 units/kg body weight) before collecting tissues. The intensity of the p-Akt and Akt immunoreactive bands was quantified (*n* = 3 per group). Values are expressed as the mean ± SEM. *p < 0.05 and **p < 0.01 vs. WT; ^#^*p* < 0.05 and ^##^*p* < 0.01 vs. time 0 or PBS

We compared systemic insulin sensitivity between 16-week-old aS6KO mice and their littermate controls. Fasting glucose levels (Fig. 3a), as well as basal and stimulated insulin levels (Fig. 3b), were significantly higher in aS6KO mice than WT mice, suggesting increased insulin resistance in aS6KO mice. Consistent with these findings, aS6KO mice exhibited higher glucose levels following intraperitoneal glucose challenge or insulin injection compared with WT mice (Fig. 3c, d).

To investigate which tissues contribute to insulin resistance in aS6KO mice, we performed a hyperinsulinemic-euglycemic clamp study. During the clamp procedure, glucose levels were maintained at ~6 mM in both groups. The glucose infusion rate (GIR), whole-body glucose turnover, and glucose uptake into skeletal muscle were significantly decreased in aS6KO mice (Fig. 3e), confirming the development of insulin resistance. The development of insulin resistance in aS6KO mice was confirmed by findings that the levels of insulin-stimulated phosphorylation of Akt (Ser473) in skeletal muscle and liver were significantly lower in aS6KO mice than WT mice (Fig. 3f). The inhibition of insulin signaling in aS6KO mice was further supported by the decrease in the phosphorylation of the Akt downstream targets (Fig. S2). These results indicate that Sirt6 deficiency in adipocytes causes systemic insulin resistance in NCD-fed mice by affecting insulin sensitivity in liver, eWAT, and skeletal muscle.

### Sirt6 deficiency increases macrophage infiltration in eWAT

**Fig. 4:**
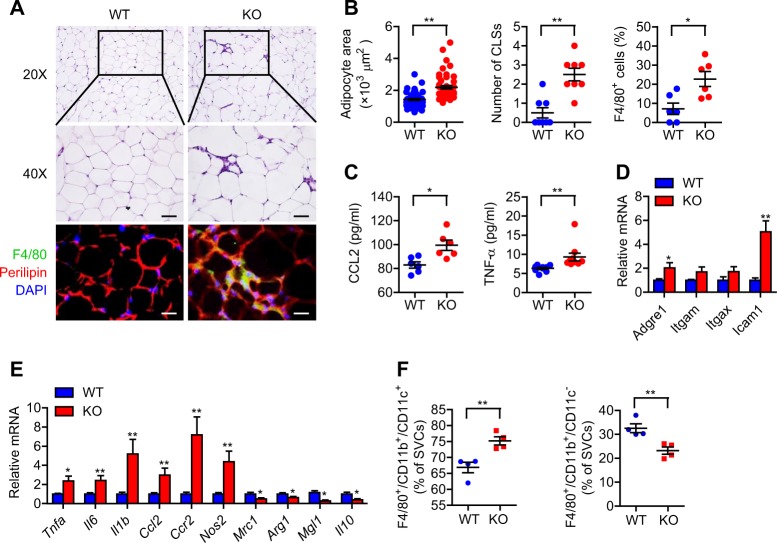
Increased adipose inflammation in aS6KO mice. **a** eWAT was stained with H&E or immunostained with an antibody against F4/80 in 16-week-old mice with NCD. Bar = 25 μm. **b** Mean surface area was measured, and the number of crown-like structures (CLSs) and F4/80^+^ cells were counted (*n* = 6 per group). **c** Plasma levels of CCL2 and TNF-α were assayed using ELISAs (*n* = 6–10 per group). The expression of macrophage infiltration- (**d**) and macrophage subtype-related genes (**e**) was determined by real-time RT-PCR (*n* = 6–10 per group). **f** The macrophage subpopulation was analyzed by FACS analysis. The M1 (F4/80^+^CD11b^+^CD11c^+^) and M2 (F4/80^+^CD11b^+^CD11c^−^) macrophage subpopulations were expressed as the percentage of stromal vascular cells (SVCs) (*n* = 5 per group). Values are expressed as the mean ± SEM. *p < 0.05 and **p < 0.01 vs. WT

Obesity is commonly associated with adipose tissue inflammation, which causes systemic insulin resistance^24^. Therefore, we next determined macrophage infiltration in WAT. Microscopic analysis of adipose tissue histology by H&E staining revealed increases in adipocyte size and the number of CLSs in aS6KO mice relative to WT mice (Fig. 4a, b). To assess macrophage infiltration into WAT, we counted cells that were immunopositive for F4/80 as a pan-marker for macrophages. The accumulation of F4/80-positive cells in eWAT was significantly higher in aS6KO mice than WT mice (Fig. 4a, b). ELISA and real-time RT-PCR analyses also confirmed the increased accumulation of macrophages and inflammation in the eWAT of aS6KO mice compared with WT mice (Fig. 4c, d). Specifically, mRNA levels of a variety of M1 macrophage genes (*Tnfa, Il6, Il1b, Ccl2, Ccr2*, and *Nos2*) were upregulated, while the mRNA levels of M2 macrophage genes (*Mrc1*, *Arg1*, *Mgl1*, and *Il10*) were downregulated in aS6KO mice (Fig. 4e). To characterize the subtypes of macrophages, we prepared SVCs from eWAT of WT and aS6KO mice and analyzed them using flow cytometry. The results revealed a higher percentage of M1-like macrophages (F4/80^+^CD11b^+^CD11c^+^) and a lower percentage of M2-like macrophages (F4/80^+^CD11b^+^CD11c^−^) in the WAT of aS6KO mice relative to WT mice (Fig. 4f & S3).

### Adipose Sirt6 regulates IL-4 production

**Fig. 5:**
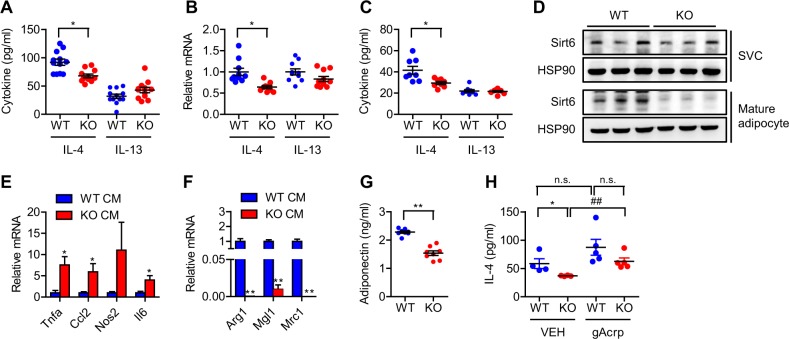
Impaired adiponectin and interleukin-4 production in aS6KO mice. **a** IL-4 and IL-13 levels in plasma were determined by ELISAs (*n* = 11–12 per group). **b** mRNA levels of *Il4* and *Il13* were determined in SVC-derived adipocytes (*n* = 9–10 per group). **c** Protein levels of IL-4 and IL-13 in culture medium of SVC-derived adipocytes were analyzed by ELISAs (*n* = 8 per group). **d** SVCs from epididymal fat pads of WT and aS6KO mice were differentiated into mature adipocytes, and Sirt6 expression was analyzed by western blotting. **e**, **f** BMMs were treated with CM from SVC-derived adipocytes for 24 h for M1 polarization and 48 h for M2 polarization. mRNA levels of M1 and M2 marker genes were assayed using real-time RT-PCR (*n* = 5–8 per group). **g** Plasma levels of adiponectin were assayed using ELISAs (*n* = 8 per group). **h** SVC-derived adipocytes were treated with 1 μg/ml globular adiponectin (gAcrp) for 18 h, and the level of secreted IL-4 in the culture medium was determined (*n* = 4–5 per group). Values are expressed as the mean ± SEM. *p < 0.05 and **p < 0.01 vs. WT; ^##^*p* < 0.01 vs. KO VEH. n.s., not significant

What is the mechanism underlying the imbalance of ATM in aS6KO mice? We first analyzed the local stimuli of M1 and M2 polarized subtypes. The plasma and tissue levels of CCL2 (also known as MCP-1), a key chemoattractant of monocytes/macrophages, were increased, whereas the level of IL-4, a well-documented inducer of M2 polarization, was significantly decreased in the aS6KO mice (Figs. 4c and 5a, & S4). However, the level of IL-13, also an inducer of M2 polarization, was not decreased. In addition, IL-4 mRNA and protein production from SVC-derived adipocytes of aS6KO mice was significantly decreased (Fig. 5b, c). Thus, we hypothesized that downregulation of IL-4 could be a possible cause of macrophage subtype imbalance in aS6KO mice. To address this issue, we analyzed the responsiveness of BMMs to CM from WT and KO adipocytes. Successful deletion of Sirt6 in KO SVC-derived adipocytes was confirmed (Fig. 5d). Treatment with CM from KO adipocytes led to an increase in M1 marker gene expression (Fig. 5e) and a decrease in M2 marker gene expression (Fig. 5f) of BMMs. Notably, we observed a decrease in adiponectin in the plasma of aS6KO mice (Fig. 5g). Because adiponectin has been reported to induce type 2 cytokines^25,26^, we then examined the effect of adiponectin treatment on IL-4 secretion from adipocytes. Addition of adiponectin to adipocytes derived from KO SVCs, but not from WT SVCs, caused a significant increase in IL-4 production (Fig. 5h), suggesting that an impairment in adiponectin production with subsequent IL-4 secretion may cause an impairment of M2 polarization in aS6KO mice.

### Visceral fat Sirt6 expression is inversely correlated with adiposity in humans

**Fig. 6:**
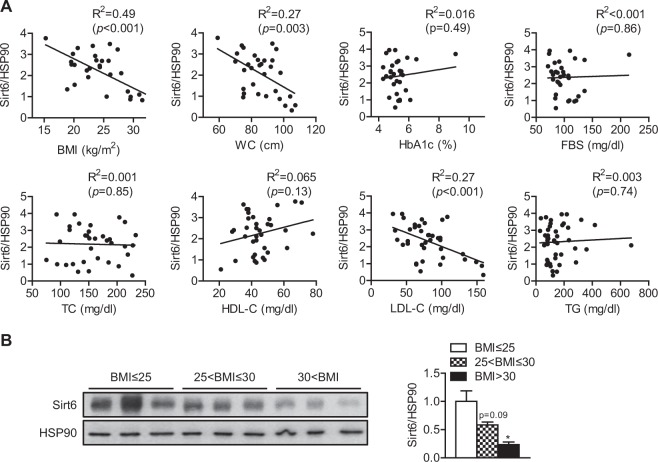
Relationship between Sirt6 expression in visceral fat and metabolic parameters in nondiabetic subjects. **a** Scatter plot of adipose Sirt6 expression and metabolic parameters. The coefficient of determination was used to compare the association of Sirt6 expression with metabolic parameters. **b** Representative western blot analysis of Sirt6 protein in visceral adipose tissue from lean (BMI ≤ 25), overweight (25 < BMI ≤ 30), and obese (30 < BMI) cohorts. The band densities were quantified. Values are expressed as the mean ± SEM. *p < 0.05 vs. the lean cohort. BMI, body mass index; WC, waist circumference; HbA1c, hemoglobin A1c; FBS, fasting blood sugar; TC, total cholesterol; HDL-C, HDL cholesterol; LDL-C, LDL cholesterol; TG, triglyceride

To determine whether Sirt6 expression in visceral adipose tissue is associated with the degree of obesity, we examined the correlation between Sirt6 expression level and physical parameters of adiposity and quantitative traits of metabolic diseases in nondiabetic subjects. Sirt6 expression in the visceral fat depot of healthy subjects correlated negatively with body mass index (BMI) and waist circumference (WC) (Fig. 6a, b). No significant correlation was observed between Sirt6 expression and other metabolic parameters in these cohorts. Stratification of the study participants into lean, overweight, and obese groups also revealed a gradual decrease in Sirt6 expression, with the highest expression in lean subjects (Fig. 6b). These data confirmed the animal data and suggest that visceral Sirt6 is a negative regulator of fat accumulation.

An inverse correlation of Sirt6 expression in visceral fat with BMI and WC was also observed in subjects with type 2 diabetes (Fig. S5). Interestingly, a negative correlation was further observed between visceral Sirt6 expression and fasting blood sugar (FBS) or hemoglobin A1c (HbA1c) levels, suggesting that low Sirt6 expression is likely to be associated with hyperglycemia as well as visceral obesity in individuals with type 2 diabetes.

## Discussion

The main finding of this study is that adipose-specific deletion of Sirt6 resulted in a decrease in adiponectin and IL-4 and an increase in CCL2 and TNF-α; all of these changes are indicative of the proinflammatory environment observed in the WAT of insulin-resistant individuals^1,27^. Consistent with these findings, histological and gene expression analyses revealed an activated proinflammatory phenotype in WAT of aS6KO mice that is reminiscent of classically activated M1 cells. Notably, IL-4 production and M2 polarization of BMMs in response to adipocyte CM were suppressed in aS6KO mice, providing evidence that Sirt6 dampens inflammation in WAT by enhancing IL-4 production and subsequent M2 polarization (Fig. S6).

We first verified the previously documented increase in fat mass induced by Sirt6 deficiency. Xiong et al.^21^ observed significant increases in body weight as well as body fat mass in *Sirt6*^*fl/fl*^:*Fabp4-Cre* mice but not in *Sirt6*^*fl/fl*^:*Adipoq-Cre* mice fed a NCD. However, Kuang et al.^20^ observed significant changes in body weight and fat mass only after HFD feeding in *Sirt6*^*fl/fl*^:*Adipoq-Cre* mice. In this study, we used *Sirt6*^*fl/fl*^:*Adipoq-Cre* mice, and consistent with a negative role of Sirt6 in lipid storage^19^, observed increases in both body weight and fat mass with NCD feeding. It is difficult to pinpoint the cause for this discrepancy, but differences in animal facility conditions or diet composition might result in different outcomes. In our study, the increased fat accumulation in aS6KO mice may be due to lower metabolic activity, as these mice displayed reduced oxygen consumption and lower energy expenditure. As we did not observe differences in food intake between WT and aS6KO mice, it is unlikely that adipose-specific deletion of *Sirt6* influenced appetite. In addition, lower lipolysis in adipocytes may cause increased adiposity in aS6KO mice.

Obese humans and genetically or diet-induced obese mice show decreased plasma and tissue levels of adiponectin, which are associated with M1-specific infiltration of macrophages in WAT and systemic insulin resistance^26,28,29^. In contrast, treatment of obese mice with insulin-sensitizing drugs restores adiponectin concentrations and induces M2 polarization of ATM^30,31^. These studies suggest that the anti-inflammatory effects of adiponectin are partly mediated by a shift in macrophages to M2 polarization. M2 polarization is mainly driven by the type 2 cytokines IL-4 and IL-13, which are produced and secreted by various cells in the adipose tissue, including adipocytes^32^. Here, we provide several lines of evidence to support the notion that adipocyte Sirt6 promotes M2 polarization by enhancing IL-4 production from adipocytes. First, IL-4 mRNA and secreted protein levels were lower in aS6KO primary adipocytes and in the plasma of aS6KO mice. Second, as a consequence, CM from aS6KO adipocytes failed to induce M2 marker genes. Third, flow cytometric and gene expression analyses showed a decrease in the M2 macrophage population in the eWAT of aS6KO mice. Notably, adiponectin treatment affected the IL-4 levels in SVC-derived adipocytes from aS6KO mice but not from WT mice, indicating that the impairment of IL-4 production observed in adipocytes from aS6KO mice is controlled by an adiponectin-dependent signaling pathway.

Because infiltration and activation of inflammatory macrophages in metabolic tissues is a key event in the pathogenesis of insulin resistance^24^, we next investigated the effects of Sirt6 deficiency on local and systemic insulin resistance. The GTT and ITT results showed glucose intolerance and insulin resistance in aS6KO mice. The hyperinsulinemic-euglycemic clamp experiment further confirmed the insulin-resistant phenotype of these mice, which showed decreased glucose infusion, glucose turnover, and insulin-stimulated glucose uptake into skeletal muscle. Muscular insulin resistance may be partly mediated by elevated TNF-α, as TNF-α has been shown to suppress glucose uptake through transcriptional downregulation of glucose transporter 4 in skeletal muscle^33^. Accordingly, insulin-stimulated Akt phosphorylation was significantly downregulated in the skeletal muscle of aS6KO mice. As discussed above, in addition to elevated TNF-α, reduced secretion of adiponectin and an increase in the M1/M2 ratio in aS6KO mice may contribute to their reduced systemic insulin sensitivity.

We recently identified myeloid Sirt6 as a key defense molecule that is required to prevent the accumulation of M1 macrophages in adipose tissue and the development of obesity and insulin resistance^24^. Suppression of the effects of M1 polarization by Sirt6 seemed to be cell autonomous, as myeloid Sirt6 was found to regulate inflammatory signaling targeting NF-κB, STAT3, and p38 mitogen-activated protein kinase. Myeloid Sirt6 KO mice fed a HFD exhibited a concomitant reduction in M2 macrophages associated with increased inflammation, suggesting that Sirt6 in myeloid-derived macrophages is crucial for M2 polarization. Together, the results of this earlier report and the current study clearly indicate that adipocyte Sirt6 in cooperation with macrophage Sirt6 is indispensable for macrophage polarization toward the M2 type. Thus, Sirt6 acts in an autocrine/paracrine manner as a common link between adipocytes and ATM to relay a dynamic interaction in the modulation of metabolic diseases.

Adipocytes are unique in their capability to produce both type 1 and type 2 cytokines. The balance of these two cytokines secreted from adipocytes and ATM dynamically determines the phenotypic features of ATM. In this study, we observed that Sirt6 deficiency in adipocytes of lean mice is sufficient to cause proinflammatory macrophage accumulation in WAT, cytokine production, and systemic insulin resistance under NCD, similar to that observed in obese mice. These findings suggest that adipose Sirt6 may be causally implicated in obesity-associated insulin resistance. In further support of this hypothesis, we found that Sirt6 expression in visceral adipose tissue of human subjects was inversely correlated with obesity parameters and insulin resistance parameters in type 2 diabetes patients. Although whether adipose Sirt6 directly or indirectly regulates adiponectin expression remains to be elucidated, our observations suggest that genetic deletion of Sirt6 triggers the M2-to-M1 transition of ATM by suppressing adiponectin and IL-4 production in adipocytes. Sirt6-mediated regulation of microenvironmental conditions in WAT, such as type 2 cytokine IL-4 production by adipocytes, may play important roles in protecting against obesity and insulin resistance.

## Supplementary information

Supplementary Information
