# Supplementary material for: Adipose sirtuin 6 drives macrophage polarization toward M2 through IL-4 production and maintains systemic insulin sensitivity in mice and humans
Source: Exp Mol Med. 2019 May 21;51(5):56. doi: 10.1038/s12276-019-0256-9 (PMC6529411; doi:10.1038/s12276-019-0256-9)
Supplement: Supplementary file 1 — Supplementary Information [file 12276_2019_256_MOESM1_ESM.pdf]

Adipose sirtuin 6 drives macrophage polarization toward M2 through IL-4 production and maintains systemic insulin sensitivity in mice and humans

Mi-Young Song[1*], Sang Hoon Kim[2*], Ga-Hee Ryoo[1], Mi-Kyung Kim[3], Hye-Na Cha[4], So-Young Park[4], Hong Pil Hwang[2], Hee Chul Yu[2], Eun Ju Bae[5¶], and Byung-Hyun Park[1¶]

[1]Department of Biochemistry and Molecular Biology, Chonbuk National University Medical School, Jeonju, Jeonbuk 54896, Republic of Korea

[2]Department of Surgery, Chonbuk National University Medical School, Jeonju, Jeonbuk 54896, Republic of Korea

[3]Research Institute of Dong-A ST Co. Ltd., Yongin, Gyeonggi 17073, Republic of Korea

[4]Department of Physiology, College of Medicine, Yeungnam University, Daegu 42415, Republic of Korea

[5]College of Pharmacy, Woosuk University, Wanju, Jeonbuk 55338, Republic of Korea

Contents

1. Supplementary Figures

2. Supplementary Tables

1. Supplementary Figures

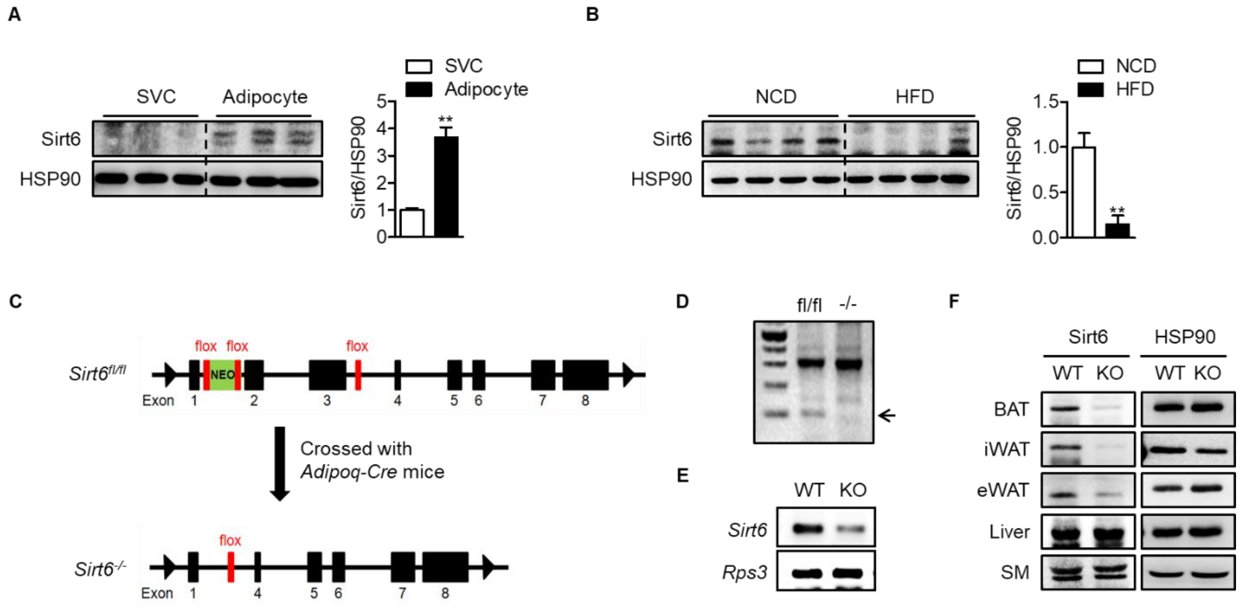

Figure S1 Generation of adipocyte-specific Sirt6 knockout mice. (A) Sirt6 protein levels in stromal vascular cells (SVCs) and adipocytes from eWAT of mice (n=3 per group). Protein density of Sirt6 was measured and normalized to that of HSP90. (B) Sirt6 protein levels in eWAT from mice fed a normal chow diet (NCD) or high-fat diet (HFD) (n=4) for 16 weeks. (C) Targeting strategy for adipocyte-specific deletion of Sirt6. (D) Genotyping of *Sirt6*$^{fl/fl}$ and *Sirt6*$^{-/-}$ mice. (E, F) mRNA and protein levels of Sirt6 in adipose and non-adipose tissues. Values are mean±SEM. $^{**}p<0.01$ vs. SVC or NCD. BAT, brown adipose tissue; iWAT, inguinal white adipose tissue; eWAT, epididymal WTA.

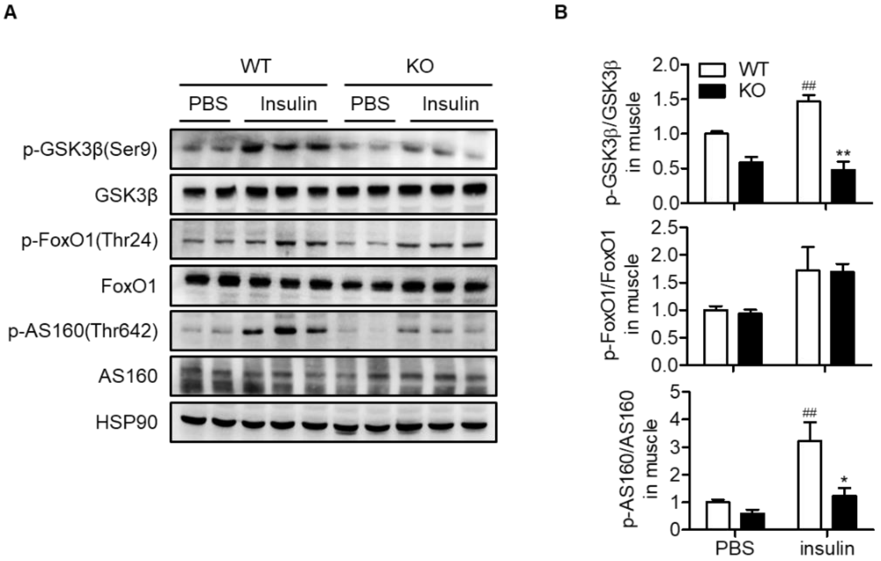

Figure S2. Downregulation of insulin signaling in aS6KO mice. (A) Western blots of the indicated total- and phosphor-proteins in skeletal muscle prepared from WT or aS6KO mice with or without insulin stimulation for 15 min. (B) The band intensities were quantified by densitometry (n=3). Values are the mean±SEM. *, p<0.05 and **, p<0.01 versus WT; $^{\#\#}$, p<0.01 versus PBS.

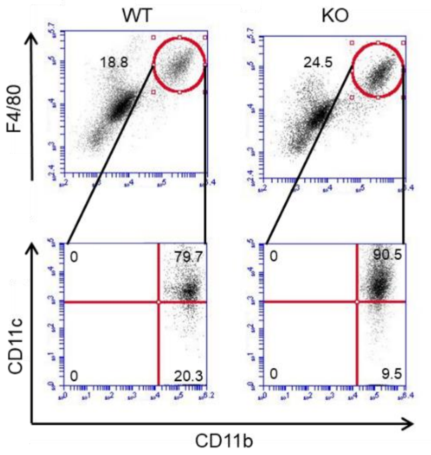

Figure S3 Flow cytometric analysis of epididymal white adipose tissue. Stromal vascular cells (SVCs) were isolated from eWAT of 10-week-old WT and aS6KO mice and stained with F4/80-FITC, CD11b-APC, and CD11c-PE. After exclusion of dead cells, the remaining cells were analyzed by flow cytometry.

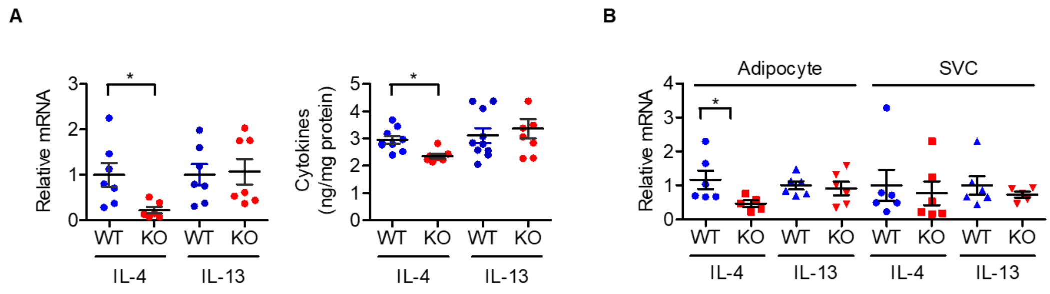

Figure S4. Cytokine expression in adipose tissues of WT and aS6KO mice. (A) mRNA and protein levels of IL-4 and IL-13 in eWAT from WT and aS6KO mice were determined by real-time RT-PCR and ELISA analyses, respectively (n=6-7 per group). (B) mRNA levels of IL-4 and IL-13 in primary adipocytes and SVCs from WT and aS6KO mice were determined by real-time RT-PCR (n=5-6 per group). Values are mean±SEM. $^{*}p<0.05$ vs. WT.

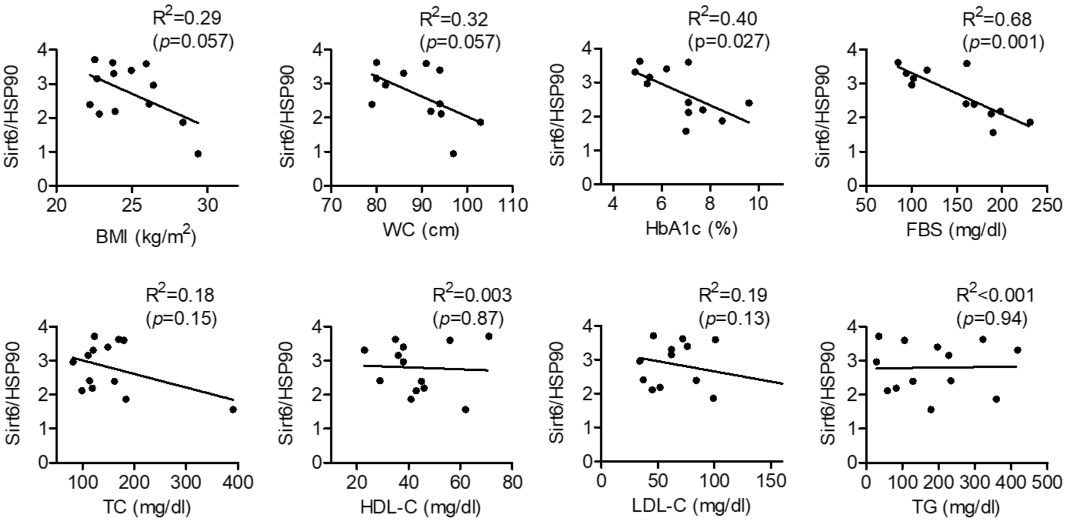

Figure S5. Relationship between Sirt6 expression in visceral fat and metabolic parameters in subjects with type 2 diabetes. Scatter plot between adipose Sirt6 expression and metabolic parameters. The coefficient of determination was used to determine the association of Sirt6 expression with metabolic parameters.

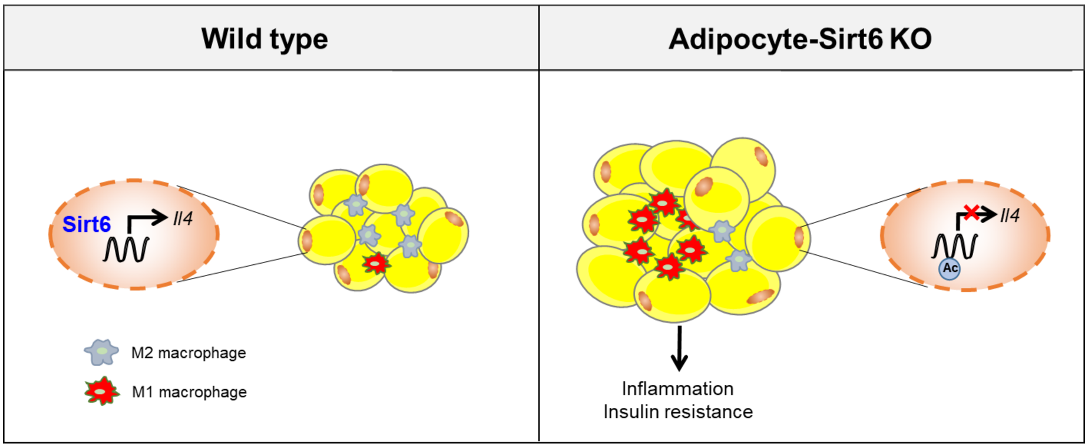

Figure S6. Proposed summary.

Supplementary Tables

Table S1. Subject characteristics

	Non-diabetic (n=31)	Diabetic (n=13)	*p* value
Gender (M/F)	26/5	12/1	0.97
Age (years)	51.10±8.49	48.54±12.31	0.50
BMI (kg/m^2)	25.06±4.92	24.84±2.28	0.88
WC (cm)	86.41±11.66	89.36±7.80	0.35
HbA1c (%)	5.20±0.54	6.82±1.58	0.0032
FBS (mg/dl)	96.73±18.33	149.08±49.48	0.0025
TC (mg/dl)	164.10±42.76	138.08±34.50	0.06
LDL-C (mg/dl)	94.68± 2.21	66.77±23.67	0.0062
HDL-C (mg/dl)	45.83±11.98	41.77±11.94	0.34
TG (mg/dl)	148.6±125.97	182.0±125.92	0.44

Values are mean±SEM. BMI, body mass index; WC, waist circumference; HbA1c, hemoglobin A1c; FBS, fasting blood sugar; TC, total cholesterol; LDL-C, LDL-cholesterol; HDL-C, HDL cholesterol; TG, triglyceride

Table S2. Sequences and accession numbers for primers (forward, FOR; reverse, REV) for real-time RT-PCR and PCR analyses

Gene	Sequences for primers	Accession NO.
Sirt6	FOR: GACACAGAGACGGCTGGAAC<br>REV: CAGACCCTCAAGCCATGTTT	NM_019812
Nos2	FOR: TTCTGTGCTGTCCCAGTGAG<br>REV: TGAAGAAAACCCCTTGTGCT	NM_010927
Adgre1	FOR: TTTCCTCGCCTGCTTCTTC<br>REV: CCCCGTCTCTGTATTCAACC	NM_010130
Il1b	FOR: GGTCAAAGGTTTGGAAGCAG<br>REV: TGTGAAATGCCACCTTTTGA	NM_008361
Tnfa	FOR: AGGGTCTGGGCCATAGAACT<br>REV: CCACCACGCTCTTCTGTCTAC	NM_013693
Il6	FOR: ACCAGAGGAAATTTTCAATAGGC<br>REV: TGATGCACTTGCAGAAAACA	NM_031168
Il10	FOR: ATGAACCGAAGCACACCATAG<br>REV: ATCAGAGAGTTGACCGCAGTTG	NM_010548
Itgam	FOR: AAGGATTCAGCAAGCCAGAA<br>REV: TAGCAGGAAAGATGGGATGG	NM_008401
Itgax	FOR: CACTCAGTGACTGCCCAAAA<br>REV: CCTCAAGACAGGACATCGCT	NM_021334
Ccl2	FOR: ATTGGGATCATCTTGCTGGT<br>REV: CCTGCTGTTCACAGTTGCC	NM_011333
Ccr2	FOR: AGCACATGTGGTGAATCCAA<br>REV: TGCCATCATAAAGGAGCCA	NM_009915
Icam1	FOR: AACAGTTCACCTGCACGGAC<br>REV: GTCACCGTTGTGATCCCTG	NM_010493
Adipoq	FOR: ACGTCATCTTCGGCATGACT<br>REV: CTCTAAAGATTGTCAGTGGATCTG	NM_009605
Mrc1	FOR: CTCGTGGATCTCCGTGACAC<br>REV: GCAAATGGAGCCGTCTGTGC	NM_008625
Mgl1	FOR: ATGATGTCTGCCAGAGAACC<br>REV: ATCACAGATTTCAGCAACCTTA	NM_010796
Arg1	FOR: TTTTTCCAGCAGACCAGCTT<br>REV: AGAGATTATCGGAGCGCCTT	NM_007482
Il4	FOR: TCGGCATTTTGAACGAGGTC<br>REV: GAAAAGCCCGAAAGAGTCTC	NM_021283
Il13	FOR: CGCAAGGCCCCCACTAC<br>REV: AAAGTGGGCTACTTCGATTTTGG	NM_008355
Rps3	FOR: AATGAACCGAAGCACACCATAG<br>REV: ATCAGAGAGTTGACCGCAGTTG	NM_010493
